# Optimal control analysis for the transmission of Nipah infection with imperfect vaccination

**DOI:** 10.1371/journal.pone.0317408

**Published:** 2025-04-16

**Authors:** Mengqi Xie, Muhammad Younas Khan, Saif ullah, Muhammad Farooq, Muhammad Bilal Riaz, Basem Al Alwan

**Affiliations:** 1 Department of ElectronicInformation Engineering, Xi’an Technological University, Xi’an, China; 2 Departmentof Mathematics, University of Peshawar, Khyber Pakhtunkhwa, Pakistan; 3 Department of Mathematics University of Science and Technology, Bannu, Pakistan; 4 IT4Innovations, VSB-Technical University of Ostrava, Ostrava, Czech Republic; 5 Jadara University Research Center, Jadara University, Irbid, Jordan; 6 ChemicalEngineering Department, College of Engineering, King Khalid University, Abha,Saudi Arabia; Kwame Nkrumah University of Science and Technology, GHANA

## Abstract

This paper presents an innovative mathematical model for assessing the dynamics and optimal control of Nipah virus (NiV) with imperfect vaccination. The model formulation considers transmissions through contaminated food and human-to-human contacts. It also incorporates the potential virus transmission through contact with a deceased body infected with NiV. Initially, the NiV model is assessed theoretically, identifying three distinct equilibrium states: the NiV-endemic equilibrium state, the NiV-free equilibrium state, and the equilibrium state involving infected flying foxes. Furthermore, the stability results of the model in the case of constant controls are thoroughly analyzed at the NiV-free equilibrium. Some of the parameters of the model are estimated based on the infected cases documented in Bangladesh from 2001 to 2017. We further perform sensitivity analysis to determine the most influential parameters and formulate effective time-dependent controls. Numerical simulations indicate the optimal course of action for eradicating the disease and provide a comparative analysis of controlling the infection under constant and time-varying interventions. The simulation confirms that the implementation of time-varying interventions is effective in minimizing disease incidence.

## 1 Introduction

NiV has become a significant paramyxovirus transmitted by bats over the last two decades, resulting in encephalitis and severe respiratory diseases in both humans and animals. NiV is included in the World Health Organization’s Blueprint list of priority viruses due to its severe nature, potential for human-to-human transmission, complex zoonotic characteristics, and the absence of effective therapeutic interventions [1]. The first identification of NiV can be traced back to a significant occurrence of respiratory sickness and encephalitis in Sungai Nipah, a village in Malaysia. This virus was carried by pigs, infecting pig farmers and causing the epidemic [2,3]. NiV outbreaks appeared in Bangladesh, Malaysia, and India from 1998 to 2018. In 2018, an outbreak was reported in Kerala, India, which is located 2,800 km away from the Bangladeshi border. NiV belongs to the Paramyxoviridae family and has the potential to cause zoonotic diseases affecting animals and humans. The virus poses a significant threat due to its ability to cause severe illnesses including brain inflammation and respiratory infections. Additionally, this virus is highly contagious and can spread rapidly through infected animals and humans. In the early first two weeks following viral exposure, an infected person can suffer symptoms such as fever, headaches, and fatigue. Confusion and disorientation arrive shortly after these symptoms, and sometimes in rare cases they can even result in a coma in less than two days [4,5].

In the current situation, no vaccine exists for NiV. However, trials in African Green monkeys using the Hendra virus vaccine are showing promise against NiV [6,7]. Further, there are no effective treatments for people or animals other than supportive care. As a result, government efforts focus on prevention by increasing public awareness. The only viable interventions include proper hospitalization, administering palliative care, and segregating affected animals to mitigate or minimize infection. Furthermore, the most optimal measure to control the incidence of NiV is to implement educational and awareness campaign to inform people about the virus and its transmission. This approach can significantly minimize the incidence of infection and its associated complications [8]. According to Halpin et al. [10], the Pteropus genus, more precisely the Pteropodidae family of flying foxes, acts as the primary host for the virus. South and Southeast Asian countries are home to more than 50 different species of Pteropus bats (Chattu et al., on Nipah virus [8]). The primary mode through which humans contract Nipah virus infection is from animals. Pteropid bats, known as flying foxes, are the natural reservoirs of this virus. Humans can acquire the virus either via direct contact with flying foxes or indirectly via contact with infectious animals. Moreover, NiV can potentially be transmitted between individuals through unhygienic contact with the body of an infectious individual prior to their funeral or burial [3,8–10].

In Bangladesh, the focus has been on surveillance and promptly identifying cases, along with implementing public health measures to prevent the transmission of the NiV. A few of these actions include putting in place quarantine regulations, putting sick animals to death, and launching awareness campaigns in public to inform people about the dangers of the illness and how to stop it from spreading. The ecological aspects of the NiV, especially its transmission patterns between bats, intermediate animals (such as pigs) and people, has been the focus of research efforts [11,12]. Eating fruits or fruit products contaminated by flying foxes can expose humans to the NiV in Bangladesh [13]. Howsoever, according to [14,15], a majority of NiV in Singapore and Malaysia are associated with direct exposure to infectious pigs. In the 2004 outbreak recorded in Bangladesh evidence of human-to-human transmission of the virus were found [16]. People who were directly exposed to the bodily fluids of NiV infected individuals or who came into interaction with contaminated objects like towels, bedsheets, etc., contracted the sickness. This includes family carers, hospital visitors, and medical professionals [17].

Computational mathematical modeling is a valuable tool for exploring the dynamics of real-life problems, including epidemics [18–22]. These models usually employ various types of differential equation systems, analyzing insights into controlling disease outbreaks [23,24]. Mathematical modeling and optimal control intervention has been recently applied for controlling various epidemics [25–31]. The integration of optimal and cost-effective analysis plays an important role in epidemiology. These analysis help in identifying the most effective strategies for eradicating an infectious disease outbreak. In recent studies [32–35], researchers applied these techniques to Lassa fever using real statistical data from Nigeria. They further studied the importance of combining mathematical modeling with real-world data to design interventions that are both effective and economical in mitigating the spread of infectious diseases.

Recent literature has seen the formulation and analysis of numerous compartmental models describing the propagation dynamics of NiV outbreaks in different regions around the globe. In [36], optimal preventive measures and controls for NiV in the Bangladesh population were identified. Several studies have explored transmission dynamics and prevention strategies. For instance, Mondal et al. [37] utilized an SEIR model to analyze the impact of new prevention techniques on disease dynamics. Shah et al. [38] developed an SEI compartmental model considering various transmission routes, including person-to-person infection and via unsafe interaction with deceased NiV patients. Some recent literature for analyzing and controlling of this infection with real statistics can be found in [39–42].

Continuing the previously discussed literature, this study utilizes computational mathematical modeling to evaluate the effects of both constant and variable intervention strategies on NiV dynamics, particularly under imperfect vaccination. It includes the normalized sensitivity results in order to indicates the most important model parameters and to formulate a control problem incorporating six control variables. It is universally believed that the use of a vaccine may be necessary to effectively curtail and eliminating infection including NiV in human populations. In our study, we focused on using a novel mathematical model to assess the impact of constant and time-dependent rates of a hypothetical imperfect vaccine on infection control. The study is organized into several sections. In the subsequent section we describe the parameters and details the formulation of the new model. Data fitting is discussed in Section 3. Section 4 includes qualitative analysis, covering equilibrium points, local and global stabilities, and the fundamental reproduction number. Section 5 focuses on sensitivity analysis of the proposed model. Optimal control analysis is the main topic of Section 6. Section 7 presents numerical simulations and graphical findings, while Section 8 provides the final conclusion.

## 2 Model formulation

This section briefly explains the procedure for developing a transmission model focusing on the dynamic features of NiV with imperfect vaccination. It is assume that the vaccinated human can catch infection. The virus primarily spreads through two modes: direct transmission from infected human to susceptible individuals, and transmission via contaminated food ingestion. The NiV model is constructed with eight distinct compartments. These compartments include the viral concentration *V*, susceptible flying foxes and human denoted by $S_{f}$ and $S_{h}$ respectively, infected flying foxes $I_{f}$, vaccinated humans $V_{h}$, infectious humans $I_{h}$, recovered humans $R_{h}$, and deceased humans due to NiV $D_{h}$. The total population of flying foxes is determined by the sum of susceptible and infected individuals, represented as $N_{f}=S_{f}+I_{f}$. Similarly, $N_{h}=S_{h}+V_{h}+I_{h}+R_{h}$ denotes the entire human population. Details for each of these compartments are provided below.

### 2.1 Modeling the dynamics of the NiV

The level of virus shedding in flying foxes that are infected is represented by the variable *p*, which decreases over time at a rate denoted by *θ*. We can express this relationship as a differential equation as:


$$\begin{array}{ccc} V^{\prime }\left(t\right)=pI_{f}-\theta V. & & \end{array}$$ (1)

### 2.2 Modeling the dynamics of flying foxes

$\Lambda_{f}$ represents the rate of recruitment and $d_{f}$ represents the natural mortality rate in the susceptible flying foxes. These vulnerable flying foxes are infected at a rate of $\beta_{1}$ upon viral exposure. The natural mortality in infected flying foxes is recorded at a rate $d_{f}$. Thus, we have the following subsystem.


$$\begin{array}{ccc} \begin{array}{c} S_{f}^{\prime }\left(t\right)=\Lambda_{f}-\frac{\beta_{1}V}{N_{f}}S_{f}-d_{f}S_{f},\\ I_{f}^{\prime }\left(t\right)=\frac{\beta_{1}V}{N_{f}}S_{f}-d_{f}I_{f}. \end{array} & & \end{array}$$ (2)

### 2.3 Modeling the dynamics of human population

The rate of food-borne viral transmission to susceptible humans is denoted by $\beta_{2}$, while the rate of transmission through interaction between infected humans and susceptible individuals is represented by $\beta_{3}$. Furthermore, coming into contact with infected corpses increases the spread of the virus at a rate of $\beta_{4}$. Using these parameters, the force of infection may be computed as follows:


$$\begin{array}{ccc} \lambda_{h}=\frac{\beta_{2}V+\beta_{3}I_{h}+\beta_{4}\kappa D_{h}}{N_{h}}, & & \end{array}$$ (3)

where a fraction denoted by *κ* represents the portion of improperly handled dead bodies contributing to the transmission of NiV. The parameter $\Lambda_{h}$ accounts for the recruitment rate of humans. The class of $S_{h}(t)$ has been added by the recovered individuals due to loss of immunity *γ* and decreases at the infection rate $\lambda_{h}$, vaccinating rate *ξ*, and the natural mortality rate $d_{h}$. The resulting differential equation is:


$$\begin{array}{ccc} S_{h}^{\prime }\left(t\right)=\Lambda_{h}-\lambda_{h}S_{h}-(d_{h}+\xi )S_{h}+\gamma R_{h}. & & \end{array}$$ (4)

Susceptible individuals are added to the vaccinated population at the vaccination rate denoted by *ξ*. The vaccinated population decreases due to the natural death rate, $d_{h}$, and the force of infection, $\eta \lambda_{h}$, where *η* represents the reduction in infection transmission achieved through vaccination. Accordingly, the dynamics of the vaccinated class are described by the following differential equation:


$$\begin{array}{ccc} V_{h}^{\prime }\left(t\right)=\xi S_{h}-\eta \lambda_{h}V_{h}-d_{h}V_{h}. & & \end{array}$$ (5)

The rate of increase in the infected human population compartment is driven by the influx of vaccinated individuals at a rate of $\eta \lambda_{h}$ and susceptible individuals at a rate of $\lambda_{h}$. Conversely, this compartment decreases due to rates $d_{1}$, *α*, and $d_{h}$, attributed to disease-induced mortality, recovery rate, and natural death, respectively.


$$\begin{array}{ccc} I_{h}^{\prime }\left(t\right)=\eta \lambda_{h}V_{h}+\lambda_{h}S_{h}-\left(\alpha +d_{1}+d_{h}\right)I_{h}, & & \end{array}$$ (6)

Recovery rate of individuals is denoted by *α*. The rates of immunity loss and death are denoted by *γ* and $d_{h}$ respectively. Thus dynamics of recovered human can be described by


$$\begin{array}{ccc} R_{h}^{\prime }\left(t\right)=\alpha I_{h}-\left(\gamma +d_{h}\right)R_{h}. & & \end{array}$$ (7)

To model the dynamics of deceased human class, it is worth mentioning that the inclusion of natural deaths from the infected class $I_{h}(t)$ into the deceased population $D_{h}(t)$ was also considered because individuals in the infected class, even if they die due to natural causes (or other unrelated reasons), still carry the infection and can contribute to its transmission. This aligns with the primary objective of our study, which is to understand and model the transmission dynamics of diseases where deceased individuals play a significant role in spreading the infection. On the other hand, individuals from other compartments who die naturally do not carry the infection and therefore do not contribute to disease transmission. Thus, the time behavior of the deceased human population is modeled by the following equation, where *ν* represents the rate at which dead human bodies are being buried.


$$\begin{array}{ccc} D_{h}^{\prime }\left(t\right)=\left(d_{1}+d_{h}\right)I_{h}-\nu D_{h}. & & \end{array}$$ (8)

The system of nonlinear differential equations that may be created by combining all of above equations can be used to describe the behavior of the NiV.


$$\begin{array}{ccc} \{\begin{array}{c} V^{\prime }\left(t\right)=pI_{f}-\theta V,\;\\ S_{f}^{\prime }\left(t\right)=\Lambda_{f}-\frac{\left(\beta_{1}V\right)}{N_{f}}S_{f}-d_{f}S_{f},\;\\ I_{f}^{\prime }\left(t\right)=\frac{\left(\beta_{1}V\right)}{N_{f}}S_{f}-d_{f}I_{f},\;\\ S_{h}^{\prime }\left(t\right)=\Lambda_{h}-\frac{\left(\beta_{2}V+\beta_{3}I_{h}+\beta_{4}\kappa D_{h}\right)}{N_{h}}S_{h}-(d_{h}+\xi )S_{h}+\gamma R_{h},\;\\ V_{h}^{\prime }\left(t\right)=\xi S_{h}-\eta \frac{\left(\beta_{2}V+\beta_{3}I_{h}+\beta_{4}\kappa D_{h}\right)}{N_{h}}V_{h}-d_{h}V_{h},\;\\ I_{h}^{\prime }\left(t\right)=\eta \frac{\left(\beta_{2}V+\beta_{3}I_{h}+\beta_{4}\kappa D_{h}\right)}{N_{h}}V_{h}+\frac{\left(\beta_{2}V+\beta_{3}I_{h}+\beta_{4}\kappa D_{h}\right)}{N_{h}}S_{h}-\left(\alpha +d_{1}+d_{h}\right)I_{h},\;\\ R_{h}^{\prime }\left(t\right)=\alpha I_{h}-\left(\gamma +d_{h}\right)R_{h},\;\\ D_{h}^{\prime }\left(t\right)=\left(d_{h}+d_{1}\right)I_{h}-\nu D_{h}.\; \end{array} & & \end{array}$$ (9)

With respective to initial conditions (ICs) as


$$\begin{array}{ccc} \{\begin{array}{c} V(0)=V_{0}\geq 0,S_{f}(0)={S{}_{f}}_{0}\geq 0,I_{f}(0)={I{}_{f}}_{0}\geq 0,S_{h}(0)={S{}_{h}}_{0}>0,\;\\ V_{h}(0)=V_{h0}\geq 0,I_{h}(0)={I{}_{h}}_{0}\geq 0,R_{h}(0)={R{}_{h}}_{0}\geq 0,D_{h}(0)={D{}_{h}}_{0}\geq 0.\; \end{array} & & \end{array}$$ (10)

**Fig 1:**
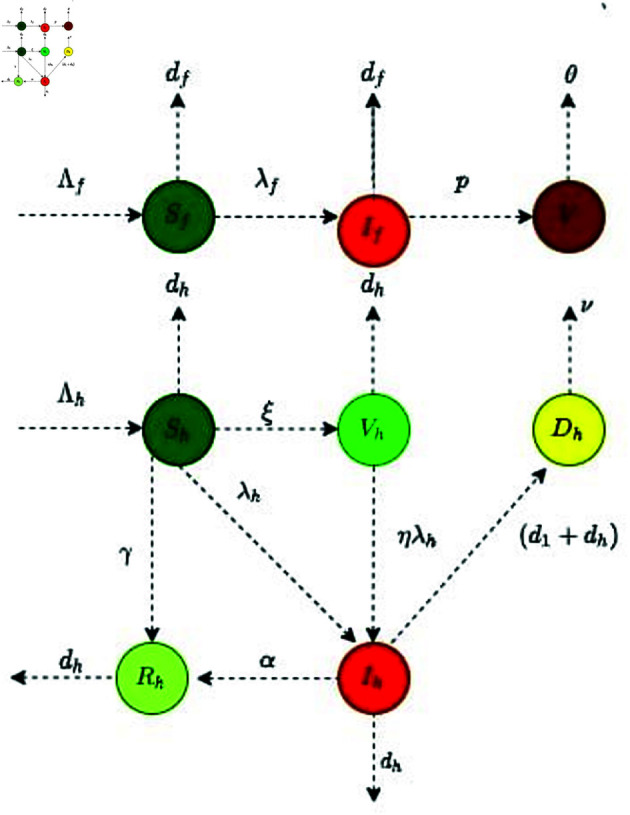
Diagram showing flow between compartments of NiV model for both flying fox and human population.

The flow among different compartments is shown by flow chart displyed in Fig 1.

## 3 Fitting and estimation of model’s parameters

In Bangladesh, this infection significantly impacted human health. Several outbreaks were recorded in Bangladesh from 2001 to 2015. The fatality rate from the illness is high, ranging from 40% to 75%.

### 3.1 Clinical overview and technique of estimating parameters

A portion of the parameters embedded in the NiV model are calculated based on clinical data. The average life expectancy in Bangladesh population is 73.57 years, as evident in [43]. The natural death rate, $d_{h}$, is calculated to be $d_{h}=\frac{1}{365\times 73.55}$ per day. The reported recovery rate from NiV is 22.457%, while the case fatality rate is particularly high, ranging from 73% to 77% [44]. As a result, the estimated recovery rate is $\alpha_{1}=0.22457$ and the estimated mortality rate caused by NiV is $d_{1}=0.7700$ [44]. The equation $\Lambda_{h}=N_{h}(0)\times d_{h}$ is used to calculate $\Lambda_{h}$, which reflects the recruitment rate of the human population. In this relation, $N_{h}(0)$ denotes the population of Bangladesh reported in 2015 [45]. Using real data and clinical information, some of the parameters values can be approximated using the same process and are listed in Table 1.

**Table 1:** Explanation and numerical values of the NiV model’s parameters.

Parameter	description	Value per day	Reference
$\Lambda_{h}$	Rate of recruiting people who are susceptible	6295 . 16	Estimated [46]
$\Lambda_{f}$	Rate of recruiting flying foxes who are susceptible	300 . 1	[47]
$d_{h}$	Human natural death rate	$\frac{1}{365\times 73.57}$	Estimated [46]
$d_{f}$	Flying foxes natural death rate	0 . 025	[47]
*ν*	Rates for burying or cremating the dead	0 . 500	[48]
*p*	Viral production rate in $I_{f}$ class	0 . 472	fitted
*θ*	Rate at which viruses vanish	0 . 091	fitted
*γ*	Getting less immune	0 . 851	[48]
$\beta_{1}$	The occurrence rate of $S_{f}$ infections	0 . 292	fitted
$\beta_{2}$	The occurrence rate of $S_{h}$ infections	0 . 650	[47]
$\beta_{3}$	Rate of interaction between $S_{h}$ and $I_{h}$	0 . 751	[48]
$\beta_{4}$	Rate of interaction between $S_{h}$ and $D_{h}$	0 . 651	[48]
*α*	Rate of recovery from a contaminated class	0 . 091	[48]
$d_{1}$	Mortality rate due to disease in the infected class	0 . 771	[49]
*κ*	$S_{h}$ contact rate with deceased	0 . 350	[47]
*η*	reduction in risk of infection due to vaccination	0 . 050	[49]
*ξ*	Vaccination rate among susceptible individuals	0 . 061	[48]

### 3.2 Validation of the model using actual data

**Fig 2:**
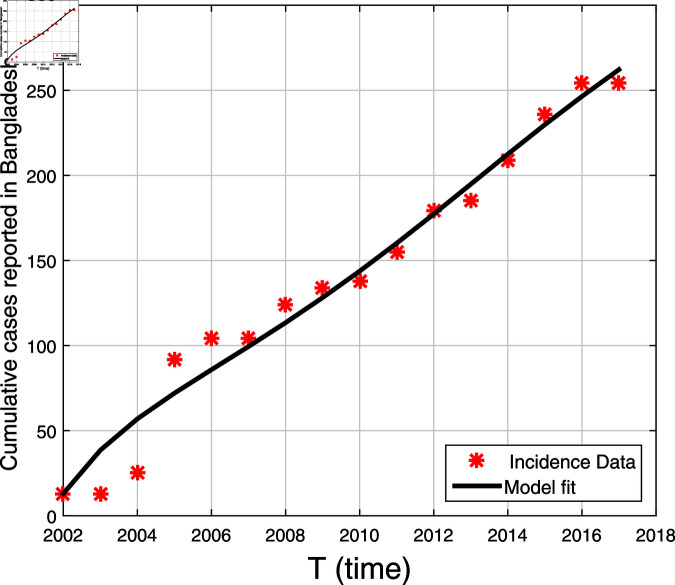
The black solid plot is a model fitting curve, while the red stars represent real data.

The fitting process of the suggested model using the actual data set provided in [45] is presented in this section. As explained in [20], we utilized the technique minimizing the residual values between the statistical and simulated data points in order to estimate the parameter values. MATLAB version R2020b is used to carry out the minimization process, and the "lsqcurvefit" technique is employed based on the subsequent relation:


$$\hat{\Omega }=argmin\overset{n}{\underset{ȷ=1}{\sum }}{\left({H_{I}}_{t_{ȷ}}-{\bar{H_{I}}}_{t_{ȷ}}\right)}^{2},$$

where *n* represents all of the data points, ${\bar{H}}_{I}t_{ȷ}$ signifies the real data, and $H_{I}t_{ȷ}$ represents the simulated cases at temporal point $t_{ȷ}$. Fig 2 shows the simulated fitted curve to the reported cases.

## 4 Analysis of the NiV model using qualitative methods

### 4.1 Feasibility of biological region

**Theorem 1.** *The model (9) with ICs stated in (10) has a consistently positive region in $\Xi =\Xi_{f}\times \Xi_{h}$, where*


$$\begin{array}{lll} \begin{array}{c} \Xi_{f}=\left\{\left(V,\;S_{f},\;I_{f}\right)\in {\mathbb{R}}_{+}^{3}:N_{f}=\frac{\Lambda_{f}}{d_{f}},V\leq \frac{p\Lambda_{f}}{d_{f}\theta }\right\},\\ \Xi_{h}=\left\{\left(S_{h},\;V_{h},\;I_{h},\;R_{h},\;D_{h}\right)\in {\mathbb{R}}_{+}^{5}:N_{h}=S_{h}+I_{h}+R_{h}+V_{h}\leq \frac{\Lambda_{h}}{\xi +d_{h}},D_{h}\leq \frac{\Lambda_{h}\left(d_{1}+d_{h}\right)}{\nu d_{h}}\right\}. \end{array} & \; & \end{array}$$

### 4.2 Investigation of threshold parameter and equilibria

The mathematical model represented by Eq (9) exhibits three points of equilibrium. The first point of equilibrium, referred to as the NiV free equilibrium point (NIFE), can be expressed as:


$$\begin{array}{ccc} E^{0}=\left(V^{0},S_{f}^{0},I_{f}^{0},S_{h}^{0},V_{h}^{0},I_{h}^{0},R_{h}^{0},D_{h}^{0}\right)=\left(0,\frac{\Lambda_{f}}{d_{f}},0,\frac{\Lambda_{h}}{c_{1}},\frac{\Lambda_{h}\xi }{c_{1}d_{h}},0,0,0\right). & & \end{array}$$ (11)

The calculation of the basic reproduction number $R_{0}$ can be determined through the application of next-generation technique [50]. The resulting expression for $R_{0}$ can be obtained by following a specific set of procedures.


$$\begin{array}{ccc} R_{0}=\max\left\{\overset{0}{\underset{f}{R}},R_{h}^{0}\right\}=\max\left\{\overset{0}{\underset{f}{R}},\overset{0}{\underset{h_{1}}{R}}+\overset{0}{\underset{h_{2}}{R}}\right\}=\max\left\{\frac{p\beta_{1}}{\theta d_{f}},\frac{\beta_{3}(d_{h}+\xi \eta )}{c_{1}c_{2}}+\frac{c_{4}\beta_{4}\kappa (d_{h}+\xi \eta )}{\nu c_{1}c_{2}}\right\}, & & \end{array}$$ (12)

where, $c_{1}=(\xi +d_{h}),$ $c_{2}=(\alpha +d_{1}+d_{h}),$ $c_{3}=(\gamma +d_{h})$ and $c_{4}=(d_{1}+d_{h}).$

For *η* = 0, (in case of perfect vaccination) the reduced reproduction number is obtain as $R_{h}^{0c}=\frac{\beta_{3}d_{h}}{c_{1}c_{2}}+\frac{c_{4}\beta_{4}\kappa d_{h}}{\nu c_{1}c_{2}}$. The reproduction number can be interpreted as follows:

- If $R_{0}=R_{f}^{0}$, then note that an increase in the rate at which the virus is produced by $I_{f}$ leads to a higher reproduction number ($R_{f}^{0}$). This, in turn, results in a higher disease incidence. Conversely, the reproduction number has an inverse relationship with *θ*. Consequently, an increased rate of virus decay leads to a reduced reproduction number, resulting in a decrease in disease incidence.
- If $R_{0}=R_{h}^{0}$, then an increase in transmission rates $\beta_{3}$ and $\beta_{4}$ leads to a higher reproduction number ($R_{h}^{0}$). Furthermore, observe that there is an inverse relationship between *ν* and the reproduction number. This shows the reduction in reproduction number with the timely and safely burial of deceased people infected with NiV.

### 4.3 Stability of the model

It is feasible to verify *NIFE*’s local stability after using the Jacobian method on it within the framework of the system (9).

** Theorem 2.** *The stability of the NIFE in system (9) is determined by the value of $R_{0}$. If $R_{0}<1$, the NIFE is stable locally asymptotically (*LAS*).*

*Proof.* The *LAS* at the NIFE can be found by examining the system (9) Jacobian matrix $J_{8}$. The Jacobian matrix at this point is as follows:


$$J_{8}=\left(\begin{array}{cccccccc} -\theta & 0 & p & 0 & 0 & 0 & 0 & 0\\ -\beta_{1} & -d_{f} & 0 & 0 & 0 & 0 & 0 & 0\\ \beta_{1} & 0 & -d_{f} & 0 & 0 & 0 & 0 & 0\\ -\beta_{2} & 0 & 0 & -(d_{h}+\xi ) & 0 & -\beta_{3} & \gamma & -\beta_{4}\kappa \\ 0 & 0 & 0 & \xi & -d_{h} & 0 & 0 & 0\\ \beta_{2} & 0 & 0 & 0 & 0 & \beta_{3}-c_{2} & 0 & \beta_{4}\kappa \\ 0 & 0 & 0 & 0 & 0 & \alpha & -(d_{h}+\gamma ) & 0\\ 0 & 0 & 0 & 0 & 0 & c_{4} & 0 & -\nu \end{array}\right).$$

In the above matrix, the eigenvalues $-d_{f},$ $-d_{h},$ $-(d_{h}+\xi ),$ and $-(d_{h}+\gamma )$ are negative. Further, we have the following sub-matrix


$$J_{4}=\left(\begin{array}{cccc} -\theta & p & 0 & 0\\ \beta_{1} & -d_{f} & 0 & 0\\ \beta_{2} & 0 & \beta_{3}-c_{2} & \beta_{4}\kappa \\ 0 & 0 & c_{4} & -\nu \end{array}\right).$$

The characteristic equation of the $J_{4}$ is given by


$$\lambda^{4}+B_{1}\lambda^{3}+B_{2}\lambda^{2}+B_{3}\lambda +B_{4}=0,$$

where,

$B_{1}=c_{2}(1-R_{h1}^{0})+(d_{f}+\theta +\nu )$,

$B_{2}=\nu c_{2}(1-R_{h}^{0})+\theta d_{f}(1-R_{f}^{0})+(\theta +d_{f})((1-R_{h1}^{0})+\nu )$,

$B_{3}=d_{f}\theta (1-R_{f}^{0})+(c_{2}(1-R_{h1}^{0})+\nu )+\nu c_{2}(1-R_{h2}^{0})(\theta +d_{f})$,

$B_{4}=d_{f}\theta \nu c_{2}(1-R_{h}^{0})(1-R_{f}^{0})$.

Obviously, the coefficients $B_{i}$, *i* = 1 , ⋯ , 4 ,  are positive for $R^{0}<1$. Further, the necessary conditions of Routh-Hurwitz can be verified. Thus, it confirm the required proof. □

### 4.4 Infected flying fox-free equilibrium state$I_{f}$

** Theorem 3.** *When, $R_{h}^{0}>1$, *∃* ⁡  a unique infected flying fox-free equilibrium (*IFFE*) point for the Nipah computational model (9).*

* Proof. *The following expression for the (*IFFE*) point can be attained by simultaneously evaluating the equations (9) in terms of the viral and human compartments, with $S_{f}=\frac{\Lambda_{f}}{d_{f}}$, $I_{f}=0$, and *V* = 0 as follows:


$$E_{h}^{**}=\left(0,\frac{\Lambda_{f}}{d_{f}},0,S_{h}^{**},V_{h}^{**},I_{h}^{**},R_{h}^{**},D_{h}^{**}\right),$$

such that


$$\begin{array}{ccc} \{\begin{array}{c} S_{h}^{**}=\frac{c_{2}c_{3}\Lambda_{h}^{**}\left(-\eta \lambda_{h}^{**}-d_{h}\right)}{\alpha \gamma \eta \xi \lambda_{h}^{**}-\left(\eta \lambda_{h}^{**}+d_{h}\right)\left(c_{2}c_{3}\left(c_{1}+\lambda_{h}^{**}\right)-\alpha \gamma \lambda_{h}^{**}\right)},\;\\ V_{h}^{**}=\frac{-c_{2}c_{3}\xi \Lambda_{h}^{**}}{\eta \lambda_{h}^{**}\left(\alpha \gamma \left(\lambda_{h}^{**}+\xi \right)-c_{2}c_{3}\left(c_{1}+\lambda_{h}^{**}\right)\right)-\mu_{h}\left(c_{2}c_{3}\left(c_{1}+\lambda_{h}^{**}\right)-\alpha \gamma \lambda_{h}^{**}\right)},\;\\ I_{h}^{**}=\frac{c_{3}\lambda_{h}^{**}\Lambda_{h}^{**}\left(\eta \left(\lambda_{h}^{**}+\xi \right)+d_{h}\right)}{\eta \lambda_{h}^{**}\left(c_{2}c_{3}\left(c_{1}+\lambda_{h}^{**}\right)-\alpha \gamma \left(\lambda_{h}^{**}+\xi \right)\right)+d_{h}\left(c_{2}c_{3}\left(c_{1}+\lambda_{h}^{**}\right)-\alpha \gamma \lambda_{h}^{**}\right)},\;\\ R_{h}^{**}=\frac{-\alpha \lambda_{h}^{**}\Lambda_{h}^{**}\left(\eta \left(\lambda_{h}^{**}+\xi \right)+d_{h}\right)}{\eta \lambda_{h}^{**}\left(\alpha \gamma \left(\lambda_{h}^{**}+\xi \right)-c_{2}c_{3}\left(c_{1}+\lambda_{h}^{**}\right)\right)-d_{h}\left(c_{2}c_{3}\left(c_{1}+\lambda_{h}^{**}\right)-\alpha \gamma \lambda_{h}^{**}\right)},\;\\ D_{h}^{**}=\frac{-c_{3}c_{4}\lambda_{h}^{**}\Lambda_{h}^{**}\left(\eta \left(\lambda_{h}^{**}+\xi \right)+d_{h}\right)}{\eta \nu \lambda_{h}^{**}\left(\alpha \gamma \left(\lambda_{h}^{**}+\xi \right)-c_{2}c_{3}\left(c_{1}+\lambda_{h}^{**}\right)\right)-\nu \mu_{h}\left(c_{2}c_{3}\left(c_{1}+\lambda_{h}^{**}\right)-\alpha \gamma \lambda_{h}^{**}\right)}.\; \end{array} & & \end{array}$$ (13)

Consider


$$\begin{array}{ccc} \lambda_{h}^{**}=\frac{\beta_{3}I_{h}^{**}+\beta_{4}\kappa D_{h}^{**}}{N_{h}^{**}}. & & \end{array}$$ (14)

Further, putting (13) in (14), we obtain


$$a_{2}{\lambda_{h}^{**}}^{2}+a_{1}\lambda_{h}^{**}+a_{0}=0;$$

with coefficient values


$$\begin{array}{rcll} a_{2} & = & \eta \nu \left(\alpha +c_{3}\right), & \\ a_{1} & = & \nu \left(\alpha +c_{3}\right)\left(\eta \xi +d_{h}\right)+c_{3}\eta \left(c_{2}\nu -\left(\beta_{3}\nu +\beta_{4}c_{4}\kappa \right)\right), & \\ a_{0} & = & c_{3}c_{1}c_{2}\nu \left(1-R_{h}^{0}\right). & \end{array}$$

The following conclusions are obtained from above

(i) There exists a unique point $E_{h}^{**}$ called *IFFE* if $a_{1}>0$ and $a_{0}<0$ if and only if $R_{h}^{0}>1$.

(ii) $E_{h}^{**}$ will be a unique point if $\left(a_{1}<0\wedge a_{0}=0\right)$  ∨  $a_{1}^{2}-4a_{0}a_{2}=0$.

(iii) The model has two *IFFE* if $a_{1}<0,a_{0}>0$ and has the positive discriminant.

(iv) An *IFFE* not available elsewhere.

Following the criteria in (i), the proposed model exhibits a unique *IFFE*. □

### 4.5 NiV-endemic equilibrium point

The Nipah virus endemic equilibrium (NVEE) can be derived by simultaneous solution of system (9) at steady state in terms of $\lambda_{h}^{**}$.


$$E^{**}=\left(V^{**},S_{f}^{**},I_{f}^{**},S_{h}^{**},V_{h}^{**},I_{h}^{**},R_{h}^{**},D_{h}^{**}\right),$$

such that


$$\begin{array}{ccc} \{\begin{array}{c} V^{**}=\frac{p\lambda_{f}^{**}\Lambda_{f}^{**}}{\theta d_{f}\left(\lambda_{f}+d_{f}\right)},\;\\ S_{f}^{**}=\frac{\Lambda_{f}^{**}}{\lambda_{f}^{**}+d_{f}},\;\\ I_{f}^{**}=\frac{\lambda_{f}^{**}\Lambda_{f}^{**}}{d_{f}\left(\lambda_{f}^{**}+d_{f}\right)},\;\\ S_{h}^{**}=\frac{c_{2}c_{3}\Lambda_{h}^{**}\left(-\eta \lambda_{h}^{**}-d_{h}\right)}{\alpha \gamma \eta \xi \lambda_{h}^{**}-\left(\eta \lambda_{h}^{**}+d_{h}\right)\left(c_{2}c_{3}\left(c_{1}+\lambda_{h}^{**}\right)-\alpha \gamma \lambda_{h}^{**}\right)},\;\\ V_{h}^{**}=\frac{-c_{2}c_{3}\xi \Lambda_{h}^{**}}{\eta \lambda_{h}^{**}\left(\alpha \gamma \left(\lambda_{h}^{**}+\xi \right)-c_{2}c_{3}\left(c_{1}+\lambda_{h}^{**}\right)\right)-\mu_{h}\left(c_{2}c_{3}\left(c_{1}+\lambda_{h}^{**}\right)-\alpha \gamma \lambda_{h}^{**}\right)},\;\\ I_{h}^{**}=\frac{c_{3}\lambda_{h}^{**}\Lambda_{h}^{**}\left(\eta \left(\lambda_{h}^{**}+\xi \right)+d_{h}\right)}{\eta \lambda_{h}^{**}\left(c_{2}c_{3}\left(c_{1}+\lambda_{h}^{**}\right)-\alpha \gamma \left(\lambda_{h}^{**}+\xi \right)\right)+d_{h}\left(c_{2}c_{3}\left(c_{1}+\lambda_{h}^{**}\right)-\alpha \gamma \lambda_{h}^{**}\right)},\;\\ R_{h}^{**}=\frac{-\alpha \lambda_{h}^{**}\Lambda_{h}^{**}\left(\eta \left(\lambda_{h}^{**}+\xi \right)+d_{h}\right)}{\eta \lambda_{h}^{**}\left(\alpha \gamma \left(\lambda_{h}^{**}+\xi \right)-c_{2}c_{3}\left(c_{1}+\lambda_{h}^{**}\right)\right)-d_{h}\left(c_{2}c_{3}\left(c_{1}+\lambda_{h}^{**}\right)-\alpha \gamma \lambda_{h}^{**}\right)},\;\\ D_{h}^{**}=\frac{-c_{3}c_{4}\lambda_{h}^{**}\Lambda_{h}^{**}\left(\eta \left(\lambda_{h}^{**}+\xi \right)+d_{h}\right)}{\eta \nu \lambda_{h}^{**}\left(\alpha \gamma \left(\lambda_{h}^{**}+\xi \right)-c_{2}c_{3}\left(c_{1}+\lambda_{h}^{**}\right)\right)-\nu \mu_{h}\left(c_{2}c_{3}\left(c_{1}+\lambda_{h}^{**}\right)-\alpha \gamma \lambda_{h}^{**}\right)}.\; \end{array} & & \end{array}$$ (15)

where,


$$\begin{array}{ccc} \lambda_{h}^{**}=\frac{\beta_{2}V^{**}+\beta_{3}I_{h}^{**}+\beta_{4}\kappa D_{h}^{**}}{N_{h}^{**}}. & & \end{array}$$ (16)

Further, putting (15) in (16), we obtain


$$b_{3}{\lambda_{h}^{**}}^{3}+b_{2}{\lambda_{h}^{**}}^{2}+b_{1}\lambda_{h}^{**}+b_{0}=0;$$

with coefficient values


$$\begin{array}{rcll} b_{3} & = & \eta \nu \left(\alpha +c_{3}\right)\Lambda_{h}, & \\ b_{2} & = & \beta_{2}\eta \Lambda_{f}\nu \left(1-R_{f}\right)\left(c_{2}c_{3}-\alpha \gamma \right)+\left(\alpha +c_{3}\right)\Lambda_{h}\nu \left(\eta \xi +d_{h}\right)+c_{3}\eta \Lambda_{h}\left(c_{2}\nu -\left(\beta_{3}\nu +\beta_{4}c_{4}\kappa \right)\right), & \\ b_{1} & = & \beta_{2}\Lambda_{f}\nu \left(1-R_{f}\right)d_{h}\left(c_{2}c_{3}-\alpha \gamma \right)+\beta_{2}\eta \Lambda_{f}\nu \left(1-R_{f}\right)\left(c_{1}c_{2}c_{3}-\alpha \gamma \xi \right)+c_{1}c_{2}c_{3}\nu \Lambda_{h}\left(1-R_{h}\right), & \\ b_{0} & = & \beta_{2}c_{1}c_{2}c_{3}\Lambda_{f}\nu \left(1-R_{f}\right)d_{h}. & \end{array}$$

Clearly, $c_{2}c_{3}-\alpha \gamma >0$ and $c_{1}c_{2}c_{3}-\alpha \gamma \xi >0$. The conclusions regarding the existence of *NVEE* can be followed from [51].

### 4.6 Global dynamics of NiV

** Theorem 4.** *The *NIFE* for the special case of the system (9) with *η* = 0, is globally asymptotically stable (GAS) if $R_{0}$ is less than 1.*

* Proof. *To obtain the required GAS of the system (9), the Lyapunov function *V* is designed as:

$V=A_{1}V+A_{2}I_{f}+A_{3}I_{h}+A_{3}D_{h},$

where, the values of $A_{1},A_{2},A_{3},$ and $A_{4}$ will be determined later.

Taking the time derivative of *V* gives us:

$V^{\prime }=A_{1}V^{\prime }+A_{2}I_{f}^{\prime }+A_{3}I_{h}^{\prime }+A_{4}D_{h}^{\prime },$

$V^{\prime }=A_{1}(pI_{f}-\theta V)+A_{2}(\frac{\beta_{1}V}{N_{f}}S_{f}-d_{f}I_{f})+A_{3}\left\{S_{h}\frac{\left(\beta_{2}V+\beta_{3}I_{h}+\beta_{4}\kappa D_{h}\right)}{N_{h}}-c_{2}I_{h}\right\}$

$+A_{4}\{c_{4}I_{h}-\nu D_{h}\}.$

Since $S_{f}\leq N_{f}$ and $S_{h}\leq N_{h}.$

$V^{\prime }\leq \{A_{2}\beta_{1}+A_{3}\beta_{2}-A_{1}\theta \}V+\{A_{1}p-A_{2}d_{f}\}I_{f}+\{A_{3}\beta_{3}+A_{4}c_{4}-c_{2}A_{3}\}I_{h}$

$+\{A_{3}\beta_{4}\kappa -A_{4}\nu \}D_{h}.$

Which can be simplified

$V^{\prime }\leq \{A_{2}\beta_{1}+A_{3}\beta_{2}-A_{1}\theta \}V+\{A_{1}p-A_{2}d_{f}\}I_{f}+\frac{1}{c_{2}A_{3}}\{\frac{A_{3}\beta_{3}+A_{4}c_{4}}{c_{2}A_{3}}-1\}I_{h}$

$+\{A_{3}\beta_{4}\kappa -A_{4}\nu \}D_{h}.$

Letting the values of $A_{1}=\frac{A_{2}\beta_{1}+A_{3}\beta_{2}}{\theta },A_{2}=\frac{A_{1}p}{d_{f}},A_{3}=d_{h},A_{4}=\frac{A_{3}\beta_{4}\kappa }{\nu }$ we arrived

$V^{\prime }\leq \frac{1}{c_{2}A_{3}}\{R_{h}^{0c}-1\}I_{h},$

where is the reduced reproduction number.

Hence, $V^{\prime }\leq 0$ if and only if $R_{h}^{0c}<1$, i.e., the Lyapunov function ($V^{\prime }$) has a negative derivative if in the reduced system’s basic reproductive number is less than one. Thus, the LaSalle’s Invariance Principle reveal that the *NIFE* of the system at special case with *η* = 0 is GAS. □

## 5 Parameters sensitivity analysis

Normalized sensitivity analysis is a useful statistical technique for identifying parameters that play key roles in the spread of a disease outbreak. We use the parametric technique [52] to perform this analysis on different model parameters affecting the basic reproductive number. Sensitivity indices are assessed to indicate the degree to which corresponding parameters affect infection incidence and control. The normalized forward sensitivity index of a specific parameter is obtained as follows:


$$\begin{array}{ccc} {\mathbb{Q}}_{x}=\frac{x}{R_{0}}\frac{\partial R_{0}}{\partial x}. & & \end{array}$$ (17)

**Fig 3:**
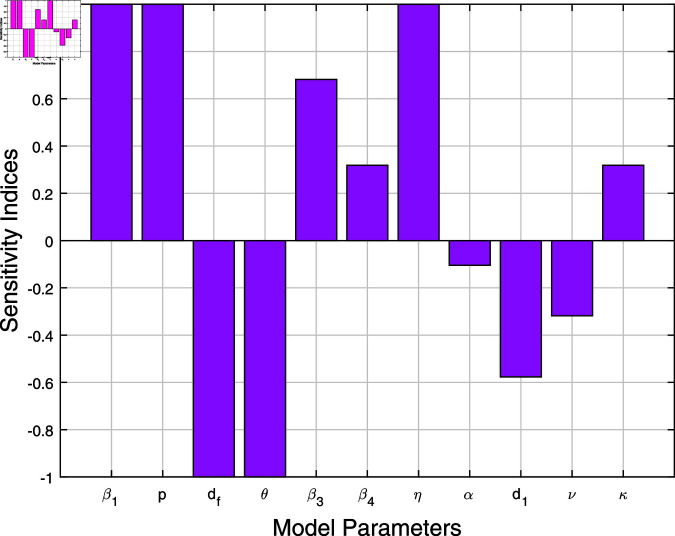
Sensitivity indices of the system’s parameters versus basic reproduction number in terms of Bar plot. Values of parameters are given in Table 1.

Using the method applied to (17), Table 2 displays the corresponding normalized sensitivity indices for $R_{h}^{0}$ and $R_{f}^{0}$ with respect to the model’s embedded parameters. The results of the sensitivity index indicate whether a parameter affects the fundamental reproduction number positively or negatively. This aids in examining both direct and inverse linkages between the threshold reproduction number and the model parameters. With positive indices, the parameters $\beta_{1}$, *p*, $\beta_{3}$, $\beta_{4}$, *η*, and *κ* directly influence $R_{h}^{0}$ and $R_{f}^{0}$. This means that $R_{h}^{0}$ and $R_{f}^{0}$ values will increase when these parameters are elevated. In contrast, the negative indices of the parameters $d_{f}$, *θ*, $d_{h}$, *α*, *ν*, and $d_{1}$ indicate an inverse relationship with the fundamental reproduction number. Consequently, $R_{h}^{0}$ and $R_{f}^{0}$ values will decrease as these parameters increase. In addition, a bar graph representing the model parameters’ sensitivity indices is displayed in Fig 3. To mitigate the occurrence of disease, this graph underscores the significance of enhancing treatment choices, reducing the rate of effective contact between susceptible and infected populations, and enhancing the effectiveness of vaccines.

**Table 2:** Sensitivity indices of model’s parameters.

Symbol	index value of $R_{\text{f}}^{0}$	Index value of $R_{h}^{0}$
$\beta_{1}$	1	
*p*	1	
$d_{f}$	-1	
*θ*	-1	
$\beta_{3}$		0.6816
$\beta_{4}$		0.3184
*η*		0.9993
*α*		-0.1046
$d_{1}$		-0.5769
*ν*		-0.3184
*κ*		0.3184

## 6 Developing the optimal control NiV model

In the NiV model shown in (9), we added six control factors to mitigate this infection at the population level. To ensure the accuracy and efficacy of the model (9), these six variable control measures, namely $v_{1}$, $v_{2}$, $v_{3}$, $v_{4}$, $v_{5}$, and $v_{6}$, have been implemented. A brief detail of each control measure is provided below, highlighting their importance in achieving the desire objectives.

- $v_{1}$: Efforts on decreasing access of fruit bats or flying foxes to date palm sap sites and other fresh food products with protective coverings
- $v_{2}$ : Implementing targeted culling of infections flying foxes, particularly in areas where outbreaks have been identified to reduce the population of infected flying foxes
- $v_{3}$ : Control the zoonotic virus transmission from infected flying foxes to people by contaminating food
- $v_{4}$ : Efferent used to reducing the risk of human-to-human transmission through personal protections
- $v_{5}$ : Adopt measures to increase the proportion of people who are vaccinated against NiV
- $v_{6}$ : Evaluate the effort necessary for health treatment of the infected human

The system structure, as previously defined by Eq (9), is presented below for the above control variables


$$\begin{array}{ccc} \{\begin{array}{c} V^{\prime }\left(t\right)=pI_{f}-\theta V,\;\\ S_{f}^{\prime }\left(t\right)=\Lambda_{f}-\frac{\left(\beta_{1}V\right)S_{f}}{N_{f}}(1-v_{1})-d_{f}S_{f},\;\\ I_{f}^{\prime }\left(t\right)=\frac{\left(\beta_{1}V\right)S_{f}}{N_{f}}(1-v_{1})-(d_{f}+v_{2})I_{f},\;\\ S_{h}^{\prime }\left(t\right)=\Lambda_{h}-\frac{\beta_{2}VS_{h}}{N_{h}}(1-v_{3})-\frac{\left(\beta_{3}I_{h}+\beta_{4}\kappa D_{h}\right)S_{h}}{N_{h}}(1-v_{4})-(d_{h}+v_{5})S_{h}+\gamma R_{h},\;\\ V_{h}^{\prime }\left(t\right)=v_{5}S_{h}-\eta \left(\frac{\beta_{2}VV_{h}}{N_{h}}(1-v_{3})+\frac{\left(\beta_{3}I_{h}+\beta_{4}\kappa D_{h}\right)V_{h}}{N_{h}}(1-v_{4})\right)-d_{h}V_{h},\;\\ I_{h}^{\prime }\left(t\right)=\frac{\beta_{2}VS_{h}}{N_{h}}(1-v_{3})+\frac{\left(\beta_{3}I_{h}+\beta_{4}\kappa D_{h}\right)S_{h}}{N_{h}}(1-v_{4})\;\\ +\eta \left(\frac{\beta_{2}VV_{h}}{N_{h}}(1-v_{3})+\frac{\left(\beta_{3}I_{h}+\beta_{4}\kappa D_{h}\right)V_{h}}{N_{h}}(1-v_{4})\right)-\left(v_{6}+d_{1}+d_{h}\right)I_{h},\;\\ R_{h}^{\prime }\left(t\right)=v_{6}I_{h}-\left(\gamma +d_{h}\right)R_{h},\;\\ D_{h}^{\prime }\left(t\right)=\left(d_{1}+d_{h}\right)I_{h}-\nu D_{h}.\; \end{array} & & \end{array}$$ (18)

Based on the baseline circumstances given in (10), our primary goal is to reduce the infected flying fox and human populations, while concurrently increasing the individuals who have recovered. This can be accomplished by putting in place the proper control mechanisms.

To obtain the best control outcome for a controlled system (18), we investigate a set of control variables $v(t)=(v_{1},v_{2},v_{3},v_{4},v_{5},v_{6})\in U$, which rely on the state variables $V,I_{f},S_{f},S_{h},I_{h},V_{h},R_{h},D_{h}$ is defined as a control period. This approach is aimed at achieving an optimal control result for the system. Control variables $v_{1},v_{2},v_{3},v_{4},v_{5}$ and $v_{6}$ are all bounded and Lebesgue measurable over the closed interval  [ 0 , 1 ]  with $U=\{(v_{1},v_{2},v_{3},v_{4},v_{5},v_{6})|0\leq v_{i}\leq 1,t\in [0,T],i=1,2,3,4,5,6\}$, where *T* is the final time. To this end, we have developed the objective functional that follows:


$$\begin{array}{ccc} J(v_{1},v_{2},v_{3},v_{4},v_{5},v_{6})=\int_{0}^{T}(A_{1}V+A_{2}I_{f}+A_{3}I_{h}+\frac{1}{2}(B_{1}v_{1}^{2}+B_{2}v_{2}^{2}+B_{3}v_{3}^{2}+B_{4}v_{4}^{2}+B_{5}v_{5}^{2}+B_{6}v_{6}^{2}))dt, & & \end{array}$$ (19)

In the objective functional (19), the controls $v_{i}$, *i* = 1 , ⋯ , 6 are quadratic because the costs of these interventions follow a nonlinear pattern. The cost of implementing control measures often does not scale linearly with the intensity of the intervention. Further, this assumption is based on published literature suggesting that the relationships between the effects of interventions and the cost of the interventions for the infected populations are nonlinear [53,54]. The objective functional (19) involves several weight constants ($A_{1}$, $A_{2}$, $A_{3}$, $B_{1}$, $B_{2}$, $B_{3}$, $B_{4}$, $B_{5}$, and $B_{6}$), which are associated with various factors such as virus and active infections in flying foxes, active infections in human patients, interaction, mortality, contamination, control, vaccinations, and treatment. Terms like $\frac{1}{2}B_{1}v_{1}^{2}$, $\frac{1}{2}B_{2}v_{2}^{2}$, $\frac{1}{2}B_{3}v_{3}^{2}$, $\frac{1}{2}B_{4}v_{4}^{2}$, $\frac{1}{2}B_{5}v_{5}^{2}$, and $\frac{1}{2}B_{6}v_{6}^{2}$ are used to express the costs associated with various control strategies. The expenses are thought to be squarely related to the relevant control measure. Finding the best control measures $\left(v_{1}^{*},v_{2}^{*},v_{3}^{*},v_{4}^{*},v_{5}^{*},v_{6}^{*}\right)$ that can reduce overall costs while maintaining the intended outcomes is the primary goal of the suggested optimal control problem.


$$\begin{array}{ccc} J(v_{1}^{*},v_{2}^{*},v_{3}^{*},v_{4}^{*},v_{5}^{*},v_{6}^{*})=\text{min}\{J(v_{1},v_{2},v_{3},v_{4},v_{5},v_{6}),(v_{1},v_{2},v_{3},v_{4},v_{5},v_{6})\in U\}. & & \end{array}$$ (20)

The control set defined for the system presented by (18)


$$\begin{array}{ccc} U=\{(v_{1},v_{2},v_{3},v_{4},v_{5},v_{6})|v_{\iota }(t)\text{ is Lebesgue measurable over }[0,1],\iota =1,..,6\}. & & \end{array}$$ (21)

The optimal controls and the necessary conditions are derived using Pontryagin’s Maximum Principle [55]. The analysis begins with a focus on the Hamiltonian *ℍ* and the Lagrangian *L* associated with the control problem formulated in  ( 18 ) − ( 20 ) . The Lagrangian for the proposed control problem is expressed as follows:


$$\begin{array}{ccc} L=A_{1}V+A_{2}I_{f}+A_{3}I_{h}+\frac{1}{2}(B_{1}v_{1}^{2}+B_{2}v_{2}^{2}+B_{3}v_{3}^{2}+B_{4}v_{4}^{2}+B_{5}v_{5}^{2}+B_{6}v_{6}^{2}). & & \end{array}$$ (22)

We seek to identify the minimum value of the aforementioned *L*. The Hamiltonian (*ℍ*) is introduced as follows


$$\begin{array}{ccc} ℍ=L+\lambda_{1}\frac{dV}{dt}+\lambda_{2}\frac{dS_{f}}{dt}+\lambda_{3}\frac{dI_{f}}{dt}+\lambda_{4}\frac{dS_{h}}{dt}+\lambda_{5}\frac{dV_{h}}{dt}+\lambda_{6}\frac{dI_{h}}{dt}+\lambda_{7}\frac{dR_{h}}{dt}+\lambda_{8}\frac{dD_{h}}{dt}, & & \end{array}$$ (23)

let, $\lambda_{1},\lambda_{2},\ldots ,\lambda_{8}$ be the associated adjoint variables. The next thing we need to do is show that the system has an optimal control (18).

** Theorem 5.** *Let $u^{*}=(v_{1}^{*},v_{2}^{*},v_{3}^{*},v_{4}^{*},v_{5}^{*},v_{6}^{*})$ and *J* be the objective functional on a given control set *U*. Then an optimal control $u^{*}\in U$ exists for system (18) with nonnegative initial conditions, such that*


$$J(v_{1}^{*},v_{2}^{*},v_{3}^{*},v_{4}^{*},v_{5}^{*},v_{6}^{*})=min_{(v_{1},v_{2},v_{3},v_{4},v_{5},v_{6})\in U}J(v_{1},v_{2},v_{3},v_{4},v_{5},v_{6}).$$

* Proof. *In order to prove the above theorem, we rely on the findings from the work of Lukes and Afeez et al. [56,57]. It is noteworthy that the objective functional *J* satisfies the required convexity for this minimization problem, and that control measures and the state values are both non-negative [57]. The set of control measures $(v_{1},v_{2},v_{3},v_{4},v_{5},v_{6})\in U$ is also, by definition, closed and convex [57]. To provide the necessary compactness for the existence of optimum control, the optimal system is likewise constrained. Finally, the integrand in Eq (22) is convex over the control set *U*, and it can be easily verified that there exist a positive constant *ϱ* > 1, as well as $\nu_{1}>0$ and $\nu_{2}>0$, which satisfy the required conditions.


$$J(v_{1},v_{2},v_{3},v_{4},v_{5},v_{6})\geq \nu_{1}{\left(|v_{1}{|}^{2}+|v_{2}{|}^{2}+|v_{3}{|}^{2}+|v_{4}{|}^{2}+|v_{5}{|}^{2}+|v_{6}{|}^{2}\right)}^{\frac{\varrho }{2}}-\nu_{2},$$

the existence of the proposed optimal control can be inferred, which serves as conclusive evidence of the proof. The detailed steps for the proof of the aforementioned condition can be easily derived from a similar NiV epidemic model [42]. □

The approach of Pontryagin’s principle has been employed to determine the optimal solution for a given problem. This involves utilizing specific conditions on *ℍ* to calculate the adjoint variables and control functions.

The Hamiltonian (*ℍ*) is subjected to the conditions mentioned earlier in order to derive the required outcomes.

**Theorem 6.** *Let $V^{*}$, $S_{f}^{*}$, $I_{f}^{*}$, $S_{h}^{*}$, $V_{h}^{*}$, $I_{h}^{*}$, $R_{h}^{*}$ and $D_{h}^{*}$ be the state solutions with associated optimal controls $v_{1}^{*}$, $v_{2}^{*}$, $v_{3}^{*}$, $v_{4}^{*}$, $v_{5}^{*}$, $v_{6}^{*}$ regarding the optimal control system denoted by (18) and (19). Afterward, the adjoint variables $\lambda_{1},\ldots ,\lambda_{8}$ satisfied the following:*

$\frac{d\lambda_{1}}{dt}=-A_{1}+\theta \lambda_{1}+\frac{S_{f}\left(1-v_{1}\right)\beta_{1}\left(\lambda_{2}-\lambda_{3}\right)}{N_{f}}+\frac{S_{h}\left(1-v_{3}\right)\beta_{2}\left(\lambda_{4}-\lambda_{6}\right)}{N_{h}}+\frac{\eta \left(1-v_{3}\right)V_{h}\beta_{2}\left(\lambda_{5}-\lambda_{6}\right)}{N_{h}},$

$\frac{d\lambda_{2}}{dt}=d_{f}\lambda_{2}+\frac{VI_{f}\left(1-v_{1}\right)\beta_{1}\left(\lambda_{2}-\lambda_{3}\right)}{N_{f}^{2}},$

$\frac{d\lambda_{3}}{dt}=-A_{2}-p\lambda_{1}+(d_{f}+v_{2})\lambda_{3}+\frac{VS_{f}\left(1-v_{1}\right)\beta_{1}\left(\lambda_{3}-\lambda_{2}\right)}{N_{f}^{2}},$

$\frac{d\lambda_{4}}{dt}=d_{h}\lambda_{4}+v_{5}\left(\lambda_{4}-\lambda_{5}\right)+\frac{1}{N_{h}}\left\{\left(V\left(1-v_{3}\right)\beta_{2}+\left(1-v_{4}\right)\left(I_{h}\beta_{3}+\kappa D_{h}\beta_{4}\right)\right)\lambda_{4}\right\}$

$+\frac{1}{N_{h}^{2}}\left\{\left(V\beta_{2}\left(v_{3}-1\right)+\left(v_{4}-1\right)\left(I_{h}\beta_{3}+\kappa D_{h}\beta_{4}\right)\right)\left(S_{h}\lambda_{4}+\eta V_{h}\lambda_{5}+\left(I_{h}+R_{h}-\eta V_{h}+V_{h}\right)\lambda_{6}\right)\right\},$

$\frac{d\lambda_{5}}{dt}=d_{h}\lambda_{5}+\frac{1}{N_{h}^{2}}\left\{\eta \left(I_{h}+R_{h}\right)\left(V\left(1-v_{3}\right)\beta_{2}+\left(1-v_{4}\right)\left(I_{h}\beta_{3}+\kappa D_{h}\beta_{4}\right)\right)\left(\lambda_{5}-\lambda_{6}\right)\right\}$

$+\frac{S_{h}}{N_{h}^{2}}\left\{2I_{h}d_{h}\lambda_{5}+2d_{h}\left(R_{h}+V_{h}\right)\lambda_{5}+\beta_{2}V\left(v_{3}-1\right)\left(\lambda_{4}-\eta \lambda_{5}+(\eta -1)\lambda_{6}\right)\right\}$

$+\frac{S_{h}}{N_{h}^{2}}\left\{+\left(v_{4}-1\right)\left(I_{h}\beta_{3}+\kappa D_{h}\beta_{4}\right)\left(\lambda_{4}-\eta \lambda_{5}+(\eta -1)\lambda_{6}\right)\right\},$

$\frac{d\lambda_{6}}{dt}=v_{6}\left(\lambda_{6}-\lambda_{7}\right)+\left(d_{1}+d_{h}\right)\left(\lambda_{6}-\lambda_{8}\right)-A_{3}$

$+\frac{\left(V\left(v_{3}-1\right)\beta_{2}+\left(v_{4}-1\right)\left(I_{h}\beta_{3}-N_{h}\beta_{3}+\kappa D_{h}\beta_{4}\right)\right)\left(S_{h}\left(\lambda_{4}-\lambda_{6}\right)+\eta V_{h}\left(\lambda_{5}-\lambda_{6}\right)\right)}{N_{h}^{2}},$

$\frac{d\lambda_{7}}{dt}=-\gamma \lambda_{4}+\left(\gamma +d_{h}\right)\lambda_{7}+\frac{1}{N_{h}^{2}}\left(\eta V_{h}\left(V\left(v_{3}-1\right)\beta_{2}+\left(v_{4}-1\right)\left(I_{h}\beta_{3}+\kappa D_{h}\beta_{4}\right)\right)\left(\lambda_{5}-\lambda_{6}\right)\right)$

$+\frac{S_{h}}{N_{h}^{2}}\left\{\left(\left(v_{4}-1\right)\left(I_{h}\beta_{3}+\kappa D_{h}\beta_{4}\right)\left(\lambda_{4}-\lambda_{6}\right)-\beta_{2}V\left(v_{3}-1\right)\left(\lambda_{4}+\lambda_{6}\right)\right)\right\},$

$\frac{d\lambda_{8}}{dt}=\frac{\kappa \left(1-v_{4}\right)\beta_{4}\left(S_{h}\left(\lambda_{4}-\lambda_{6}\right)+\eta V_{h}\left(\lambda_{5}-\lambda_{6}\right)\right)}{N_{h}}+\nu \lambda_{8}$,

*accompanied by transversality or boundary conditions*


$$\lambda_{i}(T)=0,i=1,\text{…},8.$$

*In addition to this, the optimal controls $v_{1}^{*}$, $v_{2}^{*}$, $v_{3}^{*}$, $v_{4}^{*}$, $v_{5}^{*}$ and $v_{6}^{*}$ are provided by*


$$\{\begin{array}{c} v_{1}^{*}=\text{max }\{\text{min}(\frac{1}{B_{1}}\left(\lambda_{3}-\lambda_{2}\right)\frac{\beta_{1}VS_{f}}{N_{f}},1),0\},\;\\ v_{2}^{*}=\text{max }\{\text{min}(\frac{1}{B_{2}}I_{f}\lambda_{3},1),0\},\;\\ v_{3}^{*}=\text{max }\{\text{min}(\frac{1}{B_{3}}\left[\frac{\beta_{2}\left(\lambda_{6}-\lambda_{4}\right)VS_{h}}{N_{h}}+\frac{\beta_{2}\eta \left(\lambda_{6}-\lambda_{5}\right)VV_{h}}{N_{h}}\right],1),0\},\;\\ v_{4}^{*}=\text{max }\{\text{min}(\frac{1}{B_{4}}\left[\frac{\left(\lambda_{6}-\lambda_{4}\right)S_{h}\left(\beta_{4}\kappa D_{h}+\beta_{3}I_{h}\right)}{N_{h}}+\frac{\eta \left(\lambda_{6}-\lambda_{5}\right)V_{h}\left(\beta_{4}\kappa D_{h}+\beta_{3}I_{h}\right)}{N_{h}}\right],1),0\},\;\\ v_{5}^{*}=\text{max }\{\text{min}(\frac{1}{B_{5}}\left(\lambda_{4}-\lambda_{5}\right)S_{h},1),0\},\;\\ v_{6}^{*}=\text{max }\{\text{min}(\frac{1}{B_{6}}\left(\lambda_{6}-\lambda_{7}\right)I_{h},1),0\}.\; \end{array}$$

* Proof. *The Hamiltonian function denoted by *ℍ* in Eq (23) can be utilized to calculate the optimality conditions and the adjoint system with appropriate boundary values. The adjoint equations can be derived using Pontryagin’s maximum principle, where the value of


$$\frac{d\lambda_{1}(t)}{dt}=-\frac{\partial ℍ}{\partial V},\frac{d\lambda_{2}(t)}{dt}=-\frac{\partial ℍ}{\partial S_{f}},\cdots \;,\frac{d\lambda_{8}(t)}{dt}=-\frac{\partial ℍ}{\partial D_{h}},$$

and $\lambda_{i}(T)=0$ where *i* = 1 , 2 , … , 8 are the final boundary time conditions.

To obtain the optimality variables as defined in above equation, we can employ the set of equations given below:


$$\frac{\partial ℍ}{\partial v_{1}}=0,\frac{\partial ℍ}{\partial v_{2}}=0,\frac{\partial ℍ}{\partial v_{3}}=0,\frac{\partial ℍ}{\partial v_{4}}=0,\frac{\partial ℍ}{\partial v_{5}}=0,\frac{\partial ℍ}{\partial v_{6}}=0.$$

Utilizing the control space property of *U* within the control set’s inside, we can successfully attain the desired outcome. □

## 7 Simulation and discussion

This section aims to conduct a comprehensive simulation results comparing the impact of the suggested preventive measures using time-dependent and constant controls by simulating models (18) and (9). These simulation are essential for exploring and analyzing the of different interventions in controlling the incidence of the NiV. For this propose, both the models were solved numerically using the well-known forward-backward fourth-order Runge-Kutta iterative scheme. The specific input parameters utilized in this simulation are given in Table 2. The balancing and weight constants, which are critical in the formulation of the optimal control problem, were estimated with the following values: $A_{1}=0.002$, $A_{2}=0.5$, $A_{3}=0.5$, $B_{1}=200$, $B_{2}=90$, $B_{3}=B_{6}=100$, $B_{4}=200$, and $B_{5}=50$. These values were chosen to balance the trade-offs between different objectives, such as minimizing the number of infections and the cost of interventions. Further, it is important to note that the weights used in the simulation are theoretical and were selected solely to depict the impact of the proposed control strategies suggested in this study. The aforementioned theoretical weights provide a basis for understanding how different control measures can influence the dynamics and control of NiV transmission and the potential effectiveness of time-varying versus constant control strategies on disease eradication with time.

We consider six strategies by taking different set of control variables in order to investigate efficacy of each control and to set the optimal intervention for the early eradication of the infection. It should be noted that in each scenario the sub-figure (i) displays the control profile corresponding to its occurrence. The blue dashed trajectories depict the dynamics with optimum control, whereas the red solid plots reflect the dynamics without control. There are eight classes in each section of the graphs. The graphical dynamics of each class is categories as follows: (a) Virus class; (b) Flying Foxes that are susceptible to the virus; (c) Flying Foxes that have been infected with the virus; (d) Humans who are susceptible to the virus; (e) Humans who have been vaccinated against the virus; (f) Humans who have been infected with the virus; (g) Humans who have recovered from the virus; (h) Humans who have died from the virus.

### 7.1 Strategy 1: Evaluating the effect of all control measures at the same time, i.e.,$v_{i}\neq 0,$ for*i* = 1 , . . . , 6

**Fig 4:**
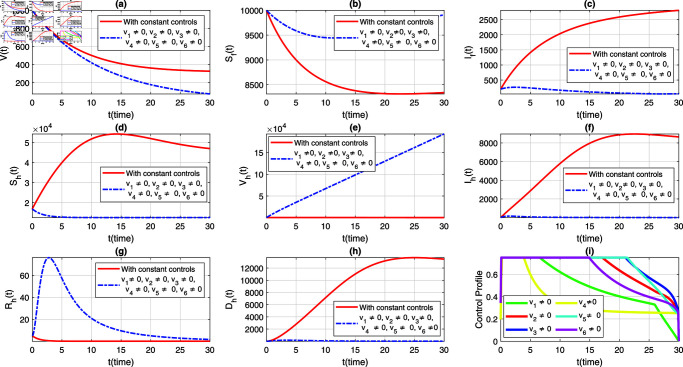
Dynamics of the NiV model under utilization of constant controls and all time-dependent controls simultaneously, where (a) *V*(*t*) (b) $S_{f}(t)$, (c) $I_{f}(t)$, (d) $S_{h}(t)$, (e) $V_{h}(t)$, (f) $I_{h}(t)$, (g) $R_{h}(t)$, (h) $D_{h}(t)$. The profile of all control variables are depicted in subplot (i).

In the first scenario, we simulate the extended model (18) to assess the population-level effects of all aforementioned time-dependent control measures on both flying foxes and humans. Simultaneously, we simulate the models (9) and (18) to provide a comparative analysis of both constant and time-varying control measures, aiming for better eradication of the infection. The graphical results of this strategy are depicted in Fig 4(a-h), with the corresponding control profile demonstrated in Fig 4(i). It is observed that with the implementation of variable controls, the viral concentration decreases significantly compared to constant control parameters, as seen in Fig 4(a). The density of infected flying foxes and humans diminishes rapidly in the presence of time-dependent controls, while in the absence of these controls (i.e., with constant control parameters), the population levels in both compartments increase, as shown in Figs 4(c) and 4(f). This comparative analysis reveals the efficacy of variable controls in the eradication of the disease. Fig 4(d) demonstrates that most of the susceptible humans are protected with optimal vaccination or other non-pharmaceutical controls. As a result, with variable controls, the population level in the susceptible class decreases while it increases dramatically in the vaccinated class, highlighting the significance of variable controls (see Figs 4(d) and 4(f)). Further, with the implementation of variable treatment interventions, the density of the recovered population increases significantly, as observed in Fig 4(g). With the suggested control measures, the population in the deceased humans class with NiV infection remains very low compared to constant control parameters (see Fig 4(h)). Additionally, the time behavior of the corresponding control profile is analyzed in Fig 4(i). From Fig 4(i), we can observe that for the effective elimination of disease, almost all controls are implemented at their maximum level in the first days and then gradually decrease over the entire course of implementation. This information can assist policymakers and health professionals in determining and implementing the appropriate and most effective control measures based on the infection’s severity and the associated containment costs.

### 7.2 Strategy 2: When $v_{1}=0,$ andother controls are hold i.e., $v_{i}\neq 0,$where, *i* = 2 , 3 , 4 , 5 , 6

**Fig 5:**
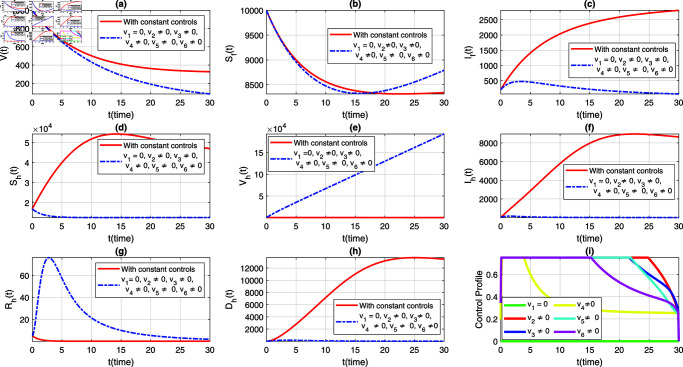
Dynamics of the NiV model under implementation of constant controls and all time-dependent controls except $v_{1}$, where (a) *V*(*t*) (b) $S_{f}(t)$, (c) $I_{f}(t)$, (d) $S_{h}(t)$, (e) $V_{h}(t)$, (f) $I_{h}(t)$, (g) $R_{h}(t)$, (h) $D_{h}(t)$. The control profile of strategy 2 is depicted in subplot (i).

In this approach, we activate all control interventions simultaneously, except for $v_{1}$, and simulate the extended NiV model (18) to assess the population-level impact on the dynamics and control of the illness. Fig 5(a)–5(h) graphically presents the dynamics of both populations to illustrate this scenario. The behavior of the corresponding control profile for this case is analyzed in Fig 5(i). As a result of this strategy, although the infected flying fox population showed a significant decrease in the presence of variable controls, they vanish over a longer period compared to the previous strategy see Fig 5(c). Further, the dynamics of the human classes remain the same as observed in the first strategy see Fig 5(d)–5(h). This indicates that the absence of $v_{1}$ has no impact on the dynamics and control of the human classes. Overall, the simulation of this case suggests that this strategy can be implemented for the early eradication of infection in the human population.

### 7.3 Strategy 3: When $v_{2}=0,$and $v_{i}\neq 0,$for *i* = 1 , 3 , 4 , 5 , 6

**Fig 6:**
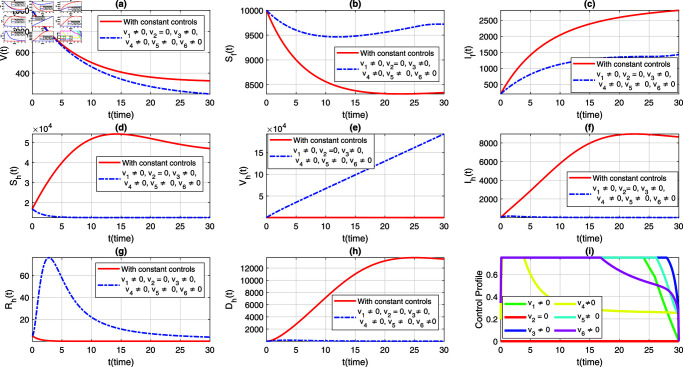
A comparative dynamics of the NiV model under implementation of constant controls and all time-dependent controls except $v_{2}$, where (a) *V*(*t*) (b) $S_{f}(t)$, (c) $I_{f}(t)$, (d) $S_{h}(t)$, (e) $V_{h}(t)$, (f) $I_{h}(t)$, (g) $R_{h}(t)$, (h) $D_{h}(t)$. The control profile of strategy 3 is depicted in subplot (i).

In the present scenario, the NiV model is simultaneously simulated by considering all controls except for the targeted culling of infected flying foxes, i.e., $v_{2}=0$. The graphical representation of this case, analyzing the variations in the dynamics of the flying fox and human populations, is depicted in Fig 6(a)–6(h). The corresponding behavior of the control profile is shown in Fig 6(i). It can be observed from the simulation that without implementing the targeted culling of infected flying foxes control ($v_{2}$), the population of infected flying foxes grows, although at a slower rate compared to the use of constant controls. This reveals that the present strategy is not effective in curtailing the infection among flying foxes (see Fig 6(c)). However, similar to previous strategies, the current set of controls is beneficial in eradicating infection incidence in humans (see Fig 6(d)–6(h)).

### 7.4 Strategy 4: When $v_{3}=0,$and $v_{i}\neq 0,$for *i* = 1 , 2 , 4 , 5 , 6

**Fig 7:**
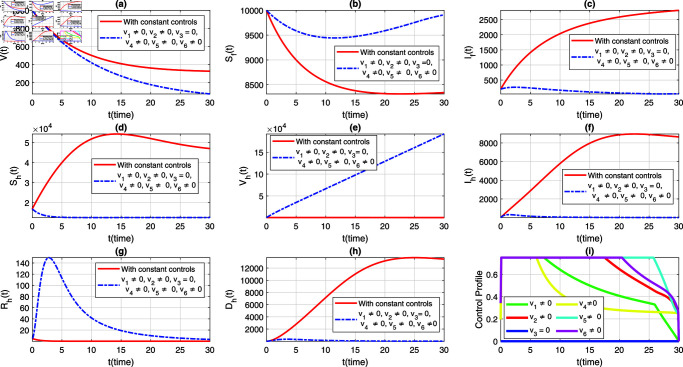
Dynamics of the NiV model under constant controls and all time-dependent controls except $v_{3}$, where (a) *V*(*t*) (b) $S_{f}(t)$, (c) $I_{f}(t)$, (d) $S_{h}(t)$, (e) $V_{h}(t)$, (f) $I_{h}(t)$, (g) $R_{h}(t)$, (h) $D_{h}(t)$. The control profile of strategy 4 is depicted in subplot (i).

This strategy implements all controls with the exception of $v_{3}$ to mitigate NiV. The simulation of this case, for the flying fox and human populations, is depicted in Fig 7(a)–7(h). The corresponding time variation in the control profile is shown in Fig 7(i). The graphical results for this case indicate outcomes similar to those of the first strategy. However, the curve for infected humans is slightly higher and takes a longer period to decline, as shown in Fig 7(f). In summery, this strategy can still be effectively implemented for the eradication of the infection in both flying fox and human populations.

### 7.5 Strategy 5: When, $v_{4}=0,$and $v_{i}\neq 0,$for *i* = 1 , 2 , 3 , 5 , 6

**Fig 8:**
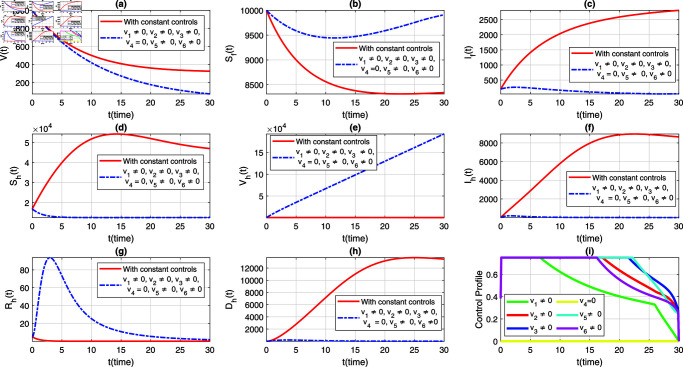
Dynamics of the NiV model under the utilization of constant controls and all time-dependent controls except $v_{1}$, where (a) *V*(*t*) (b) $S_{f}(t)$, (c) $I_{f}(t)$, (d) $S_{h}(t)$, (e) $V_{h}(t)$, (f) $I_{h}(t)$, (g) $R_{h}(t)$, (h) $D_{h}(t)$. The control profile of intervention 5 is depicted in subplot (i).

In the fifth scenario, we set the control variable $v_{4}$ to zero and implement the remaining control measures concurrently to minimize NiV infection in both populations. Fig 8(a)–8(h) shows a visual representation of the current strategy. The time behavior and intensity of the control profile over time are analyzed in Fig 8(i). Similar results are observed as in strategy 4. Although, in the presence of time-varying controls, the solution trajectory of infected humans is slightly higher compared to strategy 1 and takes a longer period to decline, as shown in Fig 8(f). However, the overall findings of this scenario indicate that it can be effectively utilized for the eradication of infection in both populations.

### 7.6 Strategy 6: In the sixth condition,$v_{i}\neq 0,$ for*i* = 1 , 2 , 3 , 4 , 5 , 6, and$v_{5}=0$

**Fig 9:**
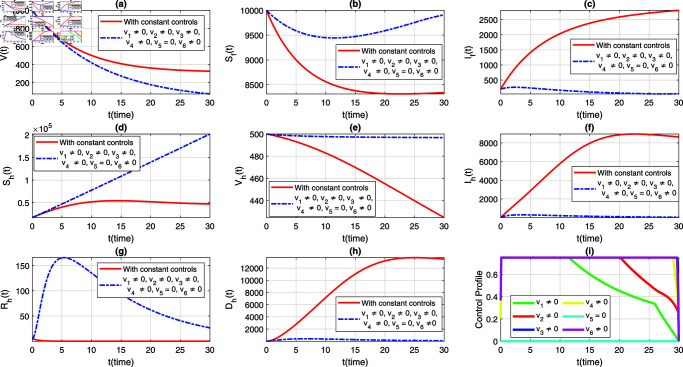
Dynamics of the NiV model under implementation of constant controls and all time-dependent controls except $v_{5}$, where (a) *V*(*t*) (b) $S_{f}(t)$, (c) $I_{f}(t)$, (d) $S_{h}(t)$, (e) $V_{h}(t)$, (f) $I_{h}(t)$, (g) $R_{h}(t)$, (h) $D_{h}(t)$. The control profile of strategy 6 is depicted in subplot (i).

In the implementation of strategy 6, all control interventions except for the vaccination control $v_{5}$ were simultaneously activated while simulating the extended NiV model (18). The resulting simulations for viral concentration and both populations are depicted in Fig 9, with subplots (a-h). Subplot 9(i) demonstrates the time variation and level of implementation of all controls except $v_{5}$. Although, this set of controls has no significant impact on the dynamics of the flying fox population as provided in strategy 1, the human population dynamics show a different trend. Without the vaccination control ($v_{5}$), the susceptible class increases rapidly, as shown in Fig 9(d). This is because of fewer people are protected with vaccination, as seen in Fig 9(e), leading to a longer time for the infected human population to decline to zero, as depicted in Fig 9(f). Therefore, we conclude that without the variable vaccination control, the implementation of the remaining controls takes comparatively longer time to completely eradicate the infection from the community.

### 7.7 In the seventh condition, $v_{i}\neq 0,$for *i* = 1 , 2 , 3 , 4 , 5,and $v_{6}=0$

**Fig 10:**
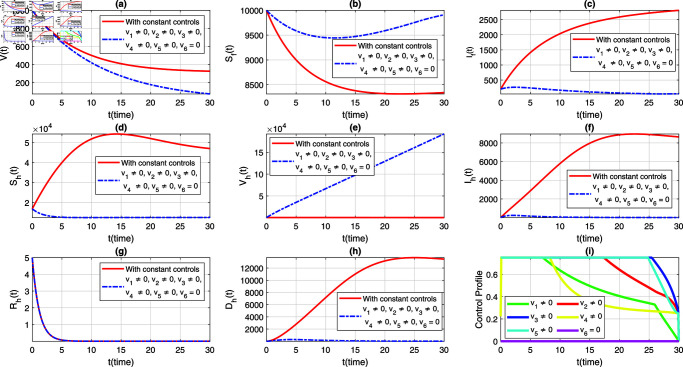
A comparative analysis of the NiV model under constant controls and all time-dependent controls except $v_{6}$, where (a) *V*(*t*) (b) $S_{f}(t)$, (c) $I_{f}(t)$, (d) $S_{h}(t)$, (e) $V_{h}(t)$, (f) $I_{h}(t)$, (g) $R_{h}(t)$, (h) $D_{h}(t)$. The control profile of strategy 7 is presented in subplot (i).

Finally, in the seventh scenario, the NiV model is simulated by considering the entire control measures except $v_{6}$ to assess the dynamics and control of the illness in both population with the help of this case. The simulations are presented in Fig 10(a)–10(h), demonstrating the dynamics of viral concentration, flying fox population, and human population, respectively. Fig 10(i) shows the corresponding control profiles for this approach. Similar to previous discussions, although in the case of variable controls, the curves for infected and deceased individuals grow comparatively higher in initial days than in strategy 1 and take slightly longer time to vanish, as seen in Fig 10(f) and 10(h).

Overall, from above simulation we claim that the first strategy is the most effective intervention and potentially reducing the incidence and impact of NiV outbreaks.

## 8 Conclusion

The present study aimed to develop a novel compartmental model for analyzing and controlling NiV in both flying fox and human populations. Unlike the existing literature, this model is formulated using imperfect vaccination in humans. Moreover, various routes of virus transmission, including human-to-human and transmission through contaminated food were incorporated into the model formulation. We examined several potential mathematical properties of the model, such as the feasible region, positivity of the solutions, the threshold number, and equilibria. The findings indicate that the model exhibits three distinct equilibrium states: one where there is no infection, another where there are no infected flying foxes, and a third where the infection is endemically present. Furthermore, the local and global stabilities of the model at the infection-free state are proven using linearization and nonlinear Lyapunov approaches. The model is validated with the statistical data of the infection in Bangladesh, and parameter estimation procedures are performed. We conducted a normalized sensitivity analysis to identify the parameters with the strongest influence on the reproduction numbers ($R_{h}^{0}$ and $R_{f}^{0}$). Based on this sensitivity analysis, we extended the model by incorporating six time-dependent control variables as detailed in the optimal control section. Furthermore, to analyze the efficacy of each control and to determine the optimal intervention for the early eradication of the infection, we presented seven intervention sceneries, each using a different set of control variables. Based on the findings from all seven strategies, we concluded that while certain scenarios show potential for controlling the infection, the optimal intervention for achieving early and effective eradication of NiV in both flying fox and human populations is the first strategy, which incorporates all control measures simultaneously. The present findings provide valuable insights for policymakers and health officials in developing effective intervention strategies for the control of the infection outbreak. In the future, to enhance the practical relevance of this study and uncover the economic feasibility, we aim to perform a cost-effective analysis of the proposed control strategies.
